# Characterization of a New Laccase from *Vibrio* sp. with pH-stability, Salt-tolerance, and Decolorization Ability

**DOI:** 10.3390/molecules28073037

**Published:** 2023-03-29

**Authors:** Jing Jiang, Jing-Ling Deng, Zhi-Gang Wang, Xiao-Yu Chen, Shu-Jie Wang, Yong-Chuang Wang

**Affiliations:** 1 School of Environmental Science and Engineering, Suzhou University of Science and Technology, Suzhou 215009, China; 2 The National Local Joint Engineering Laboratory for Municipal Sewage Resource Utilization Technology, Suzhou University of Science and Technology, Suzhou 215009, China; 3 Training Center, Qingdao Harbour Vocational & Technical College, Qingdao 266404, China

**Keywords:** laccase, pH-stability, decolorization, salt tolerance

## Abstract

Laccases have been widely used for fruit juice clarification, food modification, and paper pulp delignification. In addition, laccases exhibit remarkable performance in the degradation of toxic substances, including pesticides, organic synthetic dyes, antibiotics, and organic pollutants. Thus, the screening and development of robust laccases has attracted significant attention. In this study, *Vibrio* sp. LA is a strain capable of producing cold-adapted laccases. The laccase coding gene *L01* was cloned from this strain and expressed in *Yarrowia lipolytica*, a host with good secretion ability. The secreted L01 (approximate MW of 56,000 Da) had the activity and specific activity of 18.6 U/mL and 98.6 U/mg toward ABTS, respectively. The highest activity occurred at 35 °C. At 20 °C, L01 activity was over 70% of the maximum activity in pH conditions ranging from 4.5–10.0. Several synthetic dyes were efficiently degraded by L01. Owing to its robustness, salt tolerance, and pH stability, L01 is a promising catalytic tool for potential industrial applications.

## 1. Introduction

Laccases (EC 1.10.3.2), a class of polyphenol oxidases, are promising biocatalysts that are independent of cofactors [1]. They can oxidize various phenolic substrates and other aromatic compounds with the concurrent formation of water. Owing to their unique characteristics, laccases have been widely used in fruit juice clarification, food modification, and paper pulp delignification [1,2]. More importantly, they degrade toxic substances, including pesticides, organic synthetic dyes, antibiotics, and organic pollutants [3,4].

Laccases are already used in large scale in the textile industry. Together with low molecular weight redox-mediator compounds, laccases can generate a desired worn appearance on denim by bleaching indigo dye [2,3]. The potential use of laccase for bleaching has been investigated and this has even led to the esoteric suggestion. Recent study displayed the degradation of an azo dye, such as Sudan orange, by bacterial laccases [4].

Laccases from different organisms have been purified and characterized. The sources of these laccases include plants, insects, fungi, and bacteria [5]. The genes of these laccases have also been obtained and heterologously expressed in different hosts [6,7]. Although laccases from fungi are considered the primary choice for industrial processes, the reported fungal laccases fail to meet industrial requirements, which are restricted by low pH stability, a long fermentation period, and a low tolerance to organic solvents [6,8]. Therefore, the screening and development of robust laccases have attracted significant attention.

Laccases from bacteria generally possess a wide and suitable pH range and a large yield. Laccase-producing strains have been found in *Bacillus* sp., *Arthrospira* sp., *Achromobacter* sp., *Citrobacter* sp., *Sinorhizobium* sp., and *Klebsiella* sp. [8]. In this study, a new laccase-producing strain was screened and named *Vibrio* sp. LA. The laccase coding gene *L01* was cloned from genomic DNA and expressed in *Yarrowia lipolytica*. Recombinant laccase L01 is pH-stable and salt-tolerant; thus, it is an efficient tool for catalysis in industrial production.

## 2. Results and Discussion

### 2.1. Laccase-Producing Strain Vibrio sp. LA

Laccases are mainly found in terrestrial bacteria and fungi. To date, few marine bacteria have been shown to produce laccases [6,7,8]. Considering phenol pollution in marine environments, determining whether marine bacteria secrete laccases is of great significance [9]. The marine bacteria were isolated from 2216E plates, and then the strains were purified by streak cultivation. 24 laccase-producing strains sourced from marine bacteria were grown on 2216E medium plates containing 0.4 mM Cu^2+^ at 25 °C. With 2,2′-azino-bis-(3-ethylbenzothiazoline-6-sulfonic acid) (ABTS) as the substrate, the strain LA was detected with the highest laccase activity of 0.82 ± 0.07 U/mL at flask level (data not shown). For identification, the strain LA was subjected to 16S rDNA sequencing. BLAST alignment showed that the 16S rDNA sequence had the highest similarity (98.05%) to that of an identified strain of *Vibrio algivorus*, indicating that strain LA is a member of the genus *Vibrio*. Thus, the assigned designation was *Vibrio* sp. LA.

### 2.2. Analysis of L01 Gene Using Bioinformatics Methods

The laccase from strain LA was named L01. To obtain the *L01* gene encoding laccase secreted by *Vibrio* sp. LA, the genomic DNA of this strain was sequenced and annotated. The putative laccase-encoding gene was confirmed by catalytic region analysis. The coding sequence (CDS) starting from ATG contained 1383 bp, with a translated protein consisting 460 amino acids. It was found that the protein has a theoretical isoelectric point (pI) of 6.14 and a calculated molecular weight (MW) of 51,600 Da. In L01, the first 25 amino acids were inferred to be the signal peptide, which agreed with the extracellular location of L01. NCBI BLAST analysis revealed that L01 has a conserved domain and is a multicopper oxidase.

**Figure 1:**
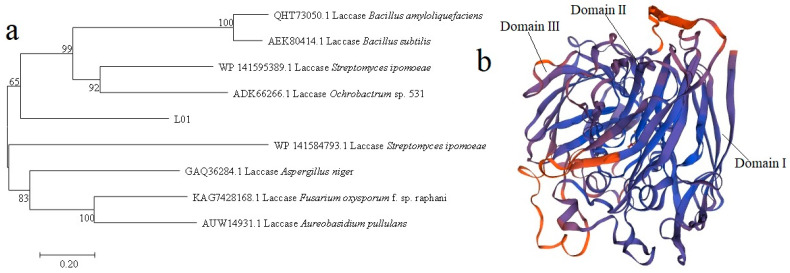
(**a**) Phylogenetic tree constructed by the neighbor-joining method with amino acid sequences of L01 and reported laccases. (**b**) Structure model of L01 by SWISS-MODEL based on the amino sequence.

**Figure 2:**
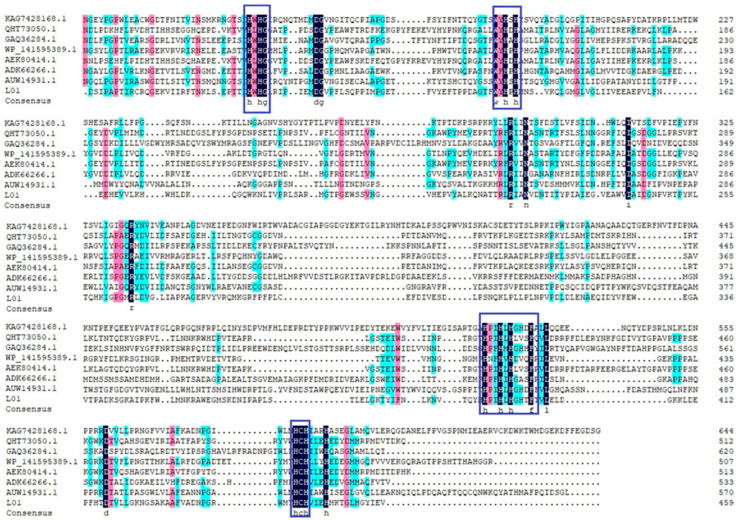
Multiple sequence alignments of L01 with other well-characterized bacterial and fungal laccases, with conserved motifs marked in blue boxes.

To further analyze the sequence characteristics of L01, we constructed a phylogenetic tree based on the amino acid sequences of L01 and laccases from other species. Clearly, the L01 groups with bacterial laccases were far from fungal laccases (Figure 1a). The closest laccases to L01 were from *Streptomyces ipomoeae* (WP 141595389.1) and *Ochrobactrum* sp. 531 (ADK66266.1) [10,11]. The structure model was constructed using the crystal structure of multicopper oxidase from *Campylobacter jejuni* (PDB: 3ZX1) as the model (Figure 1b), with a sequence similarity of 35%. L01 has three cupredoxin domains of CumA such as multicopper oxidase, with domain I (residues 36–154), domain II (residues 164–289), and domain III (residues 325–458). To further study the structural features of L01, we performed multiple sequence alignments of L01 and seven well-characterized laccases. The seven laccases were isolated from *Fusarium oxysporum* (KAG7428168.1), *Bacillus amyloliquefaciens* (QHT73050.1), *Aspergillus niger* (GAQ36284.1), *Bacillus subtilis* (AEK80414.1), *Ochrobactrum* sp. (ADK66266.1), and *Aureobasidium pullulans* (AUW14931.1). The results reflect that L01 has typical conserved motifs appearing in laccases, such as “HPXHXHGHXF”, “WYHPH”, “HWHGI”, “HPXHXHGHXF”, and “HCH” (Figure 2), which are verified copper-binding domains [12,13,14,15,16]. Thus, according to bioinformatics analysis, L01 is a typical laccase.

### 2.3. Recombinant Expression of L01 in Yarrowia lipolytica

**Figure 3:**
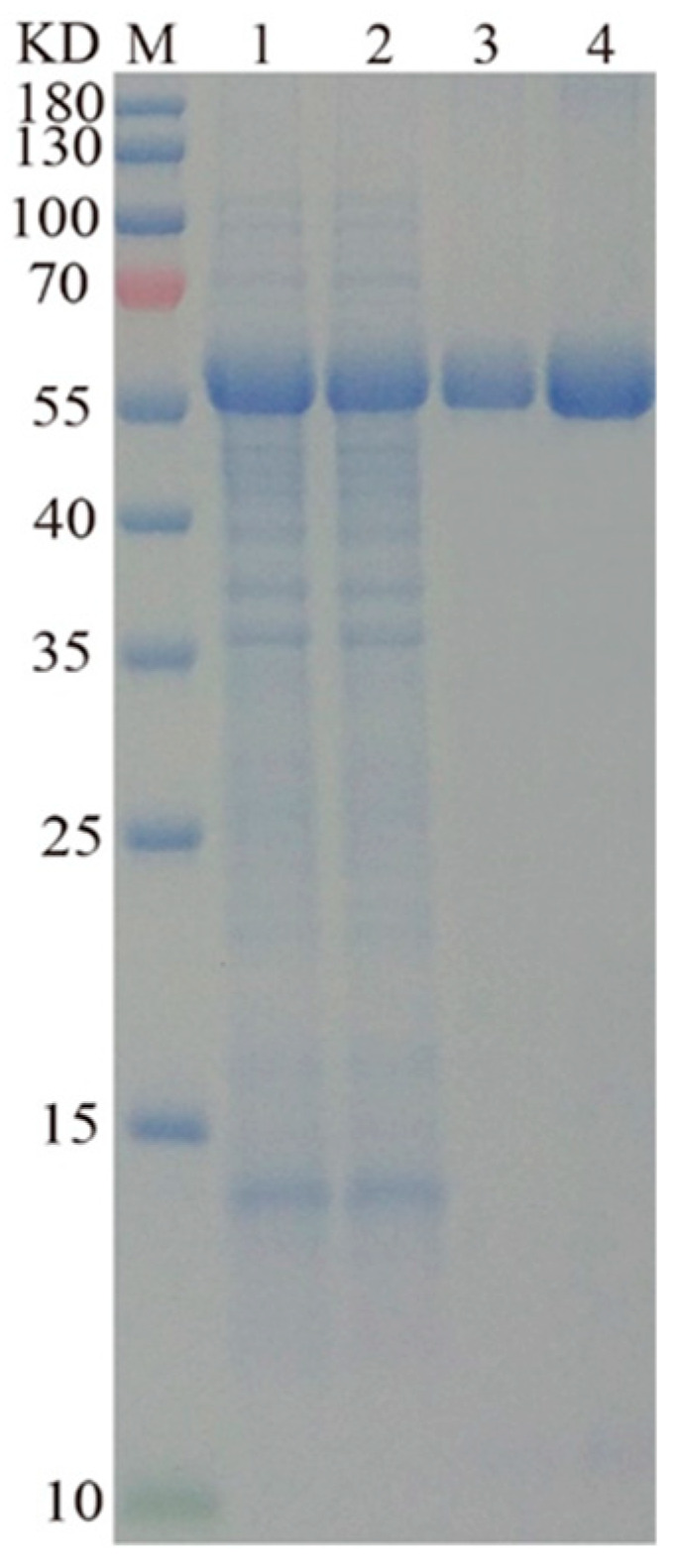
SDS-PAGE analysis of L01. Samples were loaded in different lanes. Lane M, prestained protein ladder; Lanes 1–2, the concentrated fermentation broth; Lanes 3–4, purified L01.

In previous studies, laccases were mostly expressed in *E. coli*, which is infeasible for their industrial production because of the weak secretory ability of *E. coli* and the complex post-processing procedures of laccases [6,7,17]. In this study, we expressed *L01* gene in *Yarrowia lipolytica*, a commonly used heterologous host that can produce extraordinary extracellular secretion. Many industrial enzymes have been expressed in this host with high extracellular activities [18,19,20,21]. After 60 h of incubation in the GPPB medium, the laccase secreted by the recombinant strain LA12 had an activity of up to 18.6 ± 0.6 U/mL (Figure 3), over 20-fold higher than that secreted by the wild-type strain. L01 purification was performed on the supernatant, followed by SDS-PAGE analysis. As shown in Figure 3, a clear single band was observed in lanes 3–4. The Mw of L01 was approximately 56,000, which is slightly higher than the theoretical value (52,500) of the cloned amino acids plus the His-tag. The specific activity of purified L01 toward ABTS is 98.6 ± 2.3 U/mg.

### 2.4. Effects of Temperature and pH on Activity of L01

**Figure 4:**
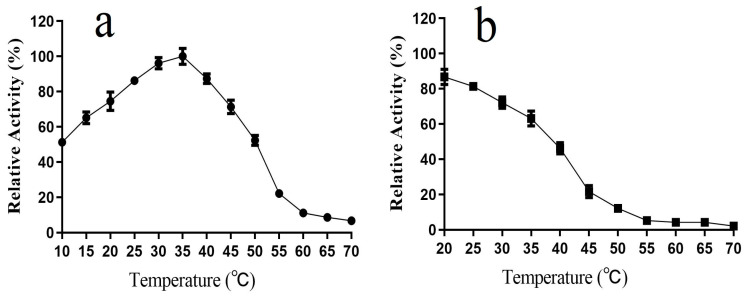
(**a**) Effect of temperature on L01 catalytic activity; (**b**) effect of temperature on L01 stability after 2 h of incubation (*n* = 3).

We investigated the enzymatic properties of purified L01 and found that its catalytic activity peaked at 35 °C toward ABTS, decreased dramatically above 50 °C, and was 51.2 ± 2.1% and 74.5 ± 5.2% of the maximum activity at 10 °C and 20 °C, respectively (Figure 4a). L01 was relatively stable at temperatures below 35 °C, and 63.1 ± 4.2% of its highest activity remained after processing at 35 °C for 2 h. However, at temperatures above 45 °C, the activity was quickly lost (Figure 4b).

Most bacterial laccases exhibit their highest activity below 40 °C. Most laccases have an activity of less than half of the highest level below 30 °C [12,22,23,24,25,26,27]. In contrast, L01 exhibits higher activity at 10 °C and 20 °C and relatively better thermostability compared to that of other laccases, which indicates that L01 is a typical cold-adapted laccase. Reactions catalyzed by cold-adapted laccases are characterized by reduced energy consumption and a low contamination risk. Thus, L01 has promising prospects as a biocatalyst candidate for industrial applications [2,6,21].

**Figure 5:**
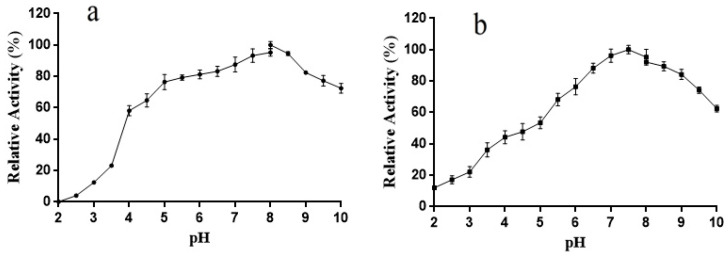
(**a**) L01 catalytic activity at different pH; (**b**) L01 stability at different pH after 4 h of incubation (*n* = 3).

The optimal pH for the activity of L01 toward ABTS was 8.0, and its activity in the range of pH 4.5–10.0 was higher than 60% of the maximum (Figure 5a). After 6 h of incubation, over 40% of the highest activity was retained at pH 4.0–10.0. Notably, in the test pH range (5.5–9.5) in this study, the activity of L01 exceeded 65% of the highest activity after incubation.

Generally, fungal laccases play a catalytic role in oxidation reactions within a narrow acidic pH range, whereas bacterial laccases prefer a relatively neutral pH [2,12,13,14,15]. Compared with the laccases reported in Table 1, L01 is stable over a much broader pH range, and pH can play a role in its catalytic activity. For example, laccases from *Aureobasidium melanogenum* and *Pseudomonas extremorientalis* are stable in narrow pH ranges of 2.8–3.6 and 7.0–10.0, respectively [12,24]. It is well recognized that pH-stable and mesophilic laccases can be generated by *Aquisalibacillus elongatus* [23]. L01 outperformed *A. elongatus* in terms of pH stability, which can contribute to the biotransformation of various phenolic substrates, especially in cases with a significant pH change.

### 2.5. L01 Activity with the Existence of Various Metal Ions and Some Compounds

**Figure 6:**
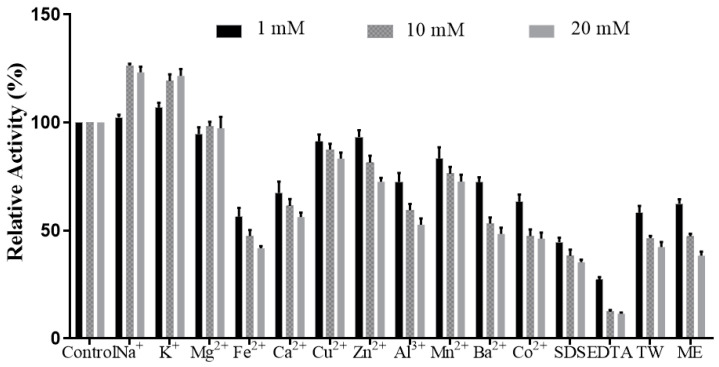
Effects of metal ions, SDS, TW, ME, and EDTA on L01 activity (*n* = 3). TW: Tween 80, ME: mercaptoethanol.

Figure 6 shows L01 activities affected by metal ions with different concentrations.

When the concentrations of K^+^ were 1 mM, 10 mM, and 20 mM, the relative activities were 106.8 ± 2.3%, 119.2 ± 3.1%, and 121.5 ± 3.2%, respectively, compared to that of the control. With the presence of Na^+^ at 10 mM or 20 mM, the relative activities were also elevated to 126.3 ± 0.9% and 123.2 ± 2.6%, respectively. Among the tested metal ions, Fe^2+^ and Ca^2+^ were detrimental to L01 activity, reducing L01 activity to 41.7 ± 1.0% and 56.1 ± 2.1% at 20 mM, respectively (Figure 6). The activity of L01 declined to 44.5 ± 2.1% and 27.2 ± 1.2% in the presence of 1 mM SDS and EDTA, respectively. With the concentrations of SDS and EDTA increased, the activities continued to decrease. 10 mM and 20 mM EDAT can reduce the activities to 12.3 ± 0.8% and 11.4 ± 0.5%. When in the presence of all metal ions at three concentrations, the activities were all above 40%. The activities were higher than those reduced by Fe^2+^ and Ca^2+^ at 10 mM and 20 mM. The above results demonstrate that L01 has a good tolerance to metal ions, and K^+^ and Na^+^ at 10 mM and 20 mM are good choices for activating agents [2,6,21]. Because it is salt-tolerant, L01 is suitable for various specific processing requirements.

### 2.6. Decolorization of Synthetic Dyes by L01

In this study, we used L01 to decolorize various synthetic dyes and found that it was effective for decolorizing several dyes. Compared with the commercial laccase from *Aspergillus oryzae*, L01 was a preferred choice for decolorizing eriochrome black T, methyl orange, and bromophenol blue. The decolorization rates for eriochrome black T, methyl orange, and bromophenol blue were 62.3 ± 3.1%, 32.1 ± 1.3%, and 46.3 ± 2.2%, respectively (Table 2). However, L01 was less satisfactory in decolorizing malachite green, crystal violet, and safranine 0, and the decolorization rates were only in the range of 10.2–15.2% within 24 h. The recombinant laccase from *Trametes hirsute* exhibited a more efficient decolorization ability for remazol brilliant blue R, whereas laccase from the basidiomycete *Coprinopsis* was efficient for indigo dye decolorization [28,29].

## 3. Materials and Methods

### 3.1. Materials, Strains, and Media

2216E plates were used to isolate bacteria from seawater samples [30]. The auxotrophic mutant yeast strain of *Y. lipolytica* and the vector pINA1312 were used for gene expression analysis. Marine sample-derived strains were cultivated in 2216E medium. A YNB plate was used to screen for the URA transformants of *Y. lipolytica*. GPPB medium (pH 6.8) was used to produce the recombinant enzyme [31].

### 3.2. Screening Laccase-Producing Strains at a Low Temperature

The isolated strains were cultivated on 2216E plates in the presence of 0.4 mM Cu^2+^ at 25 °C for 48 h to screen for potential laccase-producing strains [30]. A total of 24 laccase-producing strains sourced from marine bacteria were grown on 2216E medium plates containing 0.4 mM Cu^2+^ at 25 °C. PCR amplification was performed on the 16S rDNA of strain LA using universal primers 27F (5′-AGAGTTTGATCCTGGCTCAG-3′) and 1492R (5′-TACCTTGTTACGACTT-3′). 16S rDNA was then subjected to sequencing and BLAST alignment. After centrifugation of the culture at 5000× *g*, laccase activity of the supernatant was determined using 190 μL of 1 mM ABTS solution (in 10 mM sodium acetate buffer, pH 4.5) [32]. The reaction was conducted at 25 °C for 10 min and terminated by adding 300 μL of 10% trichloroacetic acid. One enzyme unit (U) in this study was defined as the enzyme amount required to produce an absorbance increase of one unit at 405 nm per milliliter of the mixture per minute under assay conditions. All treatments were performed in triplicate.

### 3.3. Bioinformatics Analysis of L01

To obtain the *L01* gene, the genomic DNA of LA was sent to Novogene (China) for sequencing and annotation. A putative laccase-encoding gene was identified. We analyzed the signal peptide using the online server (http://www.cbs.dtu.dk/services/SignalP-4.1/ (accessed on 11 December 2022)) and performed conserved domain analysis (https://www.ncbi.nlm.nih.gov/cdd (accessed on 11 December 2022)). The theoretical pI and Mw values were also calculated (http://web.expasy.org/compute_pi/ (accessed on 11 December 2022)). We also constructed a phylogenetic tree based on the amino acid sequences of L01 and other laccases, using MEGA version 7.0. Multiple sequence alignments were performed on L01, and laccases were characterized using DNAMAN 6.0.

### 3.4. Recombinant Expression of L01 in Yeast Host and Purification of the Recombinant Enzyme

We entrusted Synbio Technologies (Suzhou, China) to synthesize the gene *L01* with the XPR2 signal peptide after codons were optimized, and then transformed the obtained DNA fragment into a URA-strain [30]. The transformants were cultivated at 30 °C for 60 h in GPPB liquid medium and laccase activity was determined. The recombinant strain LA12 presented the highest laccase activity. After the pH of the supernatant was adjusted to 7.5, purification was conducted using a Ni–NTA column. L01 was adsorbed onto the gel, which was then washed off after loading with the imidazole solution. The purity and the Mw of L01 were estimated by SDS-PAGE analysis.

### 3.5. Impacts of Temperatures and Different pH on Activity and Stability of L01

To clarify the influence of temperature on L01 activity, we investigated a hydrolysis reaction catalyzed by L01 in glycine-NaOH buffer (10 mM, pH 8.0) at different temperatures (10–60 °C). The activity of L01 was used to determine the optimal reaction temperature. To clarify its thermal stability, we incubated purified L01 for 2 h at 10–60 °C and then detected the remaining activity at 35 °C. ABTS solutions with substrates were prepared with different pH buffers (glycine-NaOH (10 mM, pH 8.5–11.0) and Na2HPO4-citric acid (10 mM, pH 3.0–8.0)), which were used in enzymatic activity assays to determine the optimal pH for the reaction. The pH stability was estimated by detecting enzymatic activity after 4 h of incubation at 4 °C in pH buffers. The reactions were performed in triplicate.

### 3.6. L01 Activity with the Existence of Various Metal Ions and Some Compounds

High-concentration metal ions and compounds were diluted with ABTS solution to a concentration of 1 mM. L01 activity was detected in the presence of various metal ions and some compounds through reactions catalyzed by L01 at 35 °C. The reactions were performed in triplicate.

### 3.7. Decolorization of Synthetic Dyes by L01

Safranine 0 (60 mg/L), methylene blue (10 mg/L), bromophenol blue (300 mg/L), eriochrome black T (300 mg/L), crystal violet (10 mg/L), malachite green (10 mg/L), and methyl orange (10 mg/L) were prepared in a pH 5.0 solution. L01 solution and commercial laccase from *Aspergillus oryzae* (Sunson Biotech, China), with an activity of 5 U/mL was added to the dye solution. The resulting mixtures were incubated for 6 h and 24 h at 28 °C. The ODs at the maximum absorbance wavelengths were recorded using a spectrophotometer. Dyes mixed with inactivated L01 were used as controls, and their ODs at maximal absorbance wavelengths were determined using the same method [12,32]. The remaining dye was used to calculate the decolorization rate. All reactions were performed in triplicates.

## 4. Conclusions

In this study, a new cold-adapted laccase was cloned, extracellularly expressed, and characterized. Recombinant laccase L01 presented its highest activity at 35 °C and retained over 50% of its maximum activity at 10 °C and 20 °C. In addition, over 60% of the activity was maintained at pH 4.5–10.0. In addition to being pH-stable, L01 was salt-tolerant and NaCl-independent. Several synthetic dyes were efficiently degraded. L01 is an attractive biocatalyst for industrial applications owing to its robustness and unique pH stability.

## Figures and Tables

**Table 1:** Comparison of properties between L01 and reported laccases.

Source	Optimal pH/ Temperature (°C)	pH Range for Stable Protein
This study	8.0/35	4.5–10.0
*Aureobasidium melanogenum* [12]	3.2/40	2.8–3.6
*Pleurotus ostreatus* [22]	3.2/50	9.0–11.0
*Aquisalibacillus elongatus* [23]	8.0/40	5.0–10.0
*Pseudomonas extremorientalis* [24]	8.0/40–50	7.0–10.0
*Bacillus subtilis* [25]	6.8/60	5.0–7.0
*γ-Proteobacterium* [26]	6.5/55	4.0–10.0
*Pseudomonas putida* [27]	30/7.0	5.0–9.0

**Table 2:** Decolorization rates (%) of synthetic dyes by L01 and commercial laccase (*n* = 3).

Dyes	L01	Commercial Laccase
Methylene Blue	23.6 ± 2.3	15.3 ± 0.2
Malachite Green	15.2 ± 1.2	35.0 ± 2.1
Eriochrome Black T	62.3 ± 3.1	54.6 ± 1.2
Methyl Orange	32.1 ± 1.3	23.2 ± 0.8
Crystal Violet	15.2 ± 1.1	31.5 ± 1.4
Safranine 0	10.2 ± 0.4	15.3 ± 0.9
Bromophenol Blue	46.3 ± 2.2	29.0 ± 0.5

## Data Availability

Not applicable.
